# Hall conductance for open two-band system beyond rotating-wave approximation

**DOI:** 10.1038/s41598-017-16061-6

**Published:** 2017-11-24

**Authors:** W. Q. Zhang, H. Z. Shen, X. X. Yi

**Affiliations:** 1 Center for Quantum Sciences, Northeast Normal University, Changchun, 130117 China; 2 Center for Advanced Optoelectronic Functional Materials Research, and Key Laboratory for UV Light-Emitting Materials and Technology of Ministry of Education, Northeast Normal University, Changchun, 130024 China

## Abstract

The response of the open two-band system to external fields would in general be different from that of a strictly isolated one. In this paper, we systematically study the Hall conductance of a two-band model under the influence of its environment by treating the system and its environment on equal footing. In order to clarify some well-established conclusions about the Hall conductance, we do not use the rotating wave approximation (RWA) in obtaining an effective Hamiltonian. Specifically, we first derive the ground state of the whole system (the system plus the environment) beyond the RWA, then calculate an analytical expression for Hall conductance of this open system in the ground state. We apply the expression to two examples, including a magnetic semiconductor with Rashba-type spin-orbit coupling and an electron gas on a square two-dimensional lattice. The calculations show that the transition points of topological phase are robust against the environment. Our results suggest a way to the controlling of the whole system response, which has potential applications for condensed matter physics and quantum statistical mechanics.

## Introduction

Edwin. H. Hall discovered a famous phenomenon, Hall Effect, that when a conductor carrying longitudinal current was placed in a vertical magnetic field, the carriers would be pressed towards the transverse side of the conductor, which led to observed transverse voltage^1^. Its quantum counterpart–the quantum Hall effect (QHE)^2^ and the later studies on the geometric phase and the topological properties of quantum Hall states promoted the study of anomalous Hall effect, which together with the Hall and spin Hall effects completed the quantum Hall trio. The integer quantum Hall effect can be understood in the single-particle framework^3,4^ and can be characterized by the topological invariant called Chern number^5,6^ that was introduced in the QHE to explain the jump of the Hall conductance in magnetic fields. Niu, Thouless, and Wu found, for the 2D quantum Hall, a topological invariant (the first Chern number) expressed in terms of the ground-state wave function, which is valid in the presence of an arbitrary interaction and disorder^6^. Hall conductance can be represented in terms of the topological invariant (or Chern number) in the linear response theory.

The response of topological insulators (TIs) to an external weakly classical field can be expressed in terms of Kubo formula^7^, which predicts quantized Hall conductivity of the quantum Hall family. TIs were theoretically predicted and have been experimentally discovered^8–12^. In contrast to ordinary band insulators, TIs are a broad class of unconventional materials that are insulating in the interior but conductive along edges. Although time-reversal (TR) invariance is essential in the quantum spin Hall (QSH) insulator, there is a TR symmetry-breaking state of matter which is closely related to the QSH insulator: the quantum anomalous Hall (QAH) insulator^12,13^. The quantum spin Hall effect can be understood as two copies of the quantum anomalous Hall effect; each breaks TR symmetry while the whole system remains invariant under TR. In the past few years, scientific community has paid much attention to these topological materials due to their specific features. Most recently, efforts have made to investigate topological materials for open systems. For example, zero-temperature Hall conductance subjected to decoherence^14^, topological order by dissipation^15–17^, density-matrix Chern insulator subjected to thermal noise^18,19^, topological phases induced photocurrent^20,21^, and optical Hall conductivity^22–25^. Taking the quantum nature of the external field into account, a Hall conductance to characterize the linear response of a quantum system to a single-mode quantized electronmagnetic field was also defined and explored^26^. However, most of these studies are based on the reduced density matrix of the system, hence the environment is traced out before proceeding to analyze the response of the system. In many works on the linear response theory of open quantum systems, it is assumed that the effective Hamiltonian is obtained within the RWA^19,27^. This motivates us to develop a quantum response theory^28,29^ for open system including the influence of the counter-rotating terms on Hall conductance, and apply it to study two-band model subjected to environments.

For this purpose, we will study the response of the open two-band system to the external field by calculating the Hall conductance when the whole system is in its ground state. The total system consists of a two-band model and its environment described by multi-mode quantized fields. We will consider the multi-mode fields entering the system via a vector potential^30–34^.

In this paper, we will derive the ground state of the whole system without the RWA, and then calculate the Hall conductance when the whole system is in its ground state. This approach is not limited to any specific systems, as long as they can be described by the two-band model. We take the tight-binding model and an electron gas on a square two-dimensional lattice as examples to illustrate the theory. The calculations show that while the transition points of topological phase are robust against the environment, the Hall conductance is smaller than that without the environment.

## Methods

### System and Effective Hamiltonian

The model we study is a generic two-band Hamiltonian,

1 $$\begin{array}{l}{H}_{0}(\vec{k})=\vec{d}(\vec{k})\cdot \vec{\sigma }+\varepsilon (\vec{k})\cdot {\rm{I}},\end{array}$$

where I is the $2\times 2$ identity matrix, $\sigma =({\sigma }_{x},\,{\sigma }_{y},\,{\sigma }_{z})$ are Pauli matrices and $\vec{k}=({k}_{x},\,{k}_{y})$ stands for the Bloch wave vector of the electron, $\varepsilon (\vec{k})$ and $\vec{d}(\vec{k})$ depend on the materials under study and determine its band structure. The term $\varepsilon (\vec{k})$ is just a shift of zero energy level, and then can be neglected^13^ for simplicity. The two band may represent different physical degrees of freedom, for example, if they stand for the components of electron with spin 1/2, $\vec{d}(\vec{k})$ are the momentum-dependent coefficients which describe the spin-orbit interactions and exchange interaction of magnetic impurities; and if they are equal to the orbital degree of freedoms, then $\vec{d}(\vec{k})$ describes the hybridization between bands^35^.

The system in a field can still be described by the two-band model by changing the crystal momentum, $\vec{k} \rightarrow (\vec{k}-\frac{e}{\hslash }\vec{A})$, where the electromagnetic field is represented by the vector potential $\vec{A}$. Considering an electromagnetic environment, the vector potential can be written as $\vec{A}=-{\sum }_{n}{g}_{n}{\vec{\varepsilon }}_{k}({b}_{n}+{b}_{n}^{\dagger })$ ^36^, ${\vec{\varepsilon }}_{k}=({\vec{e}}_{x},\,{\vec{e}}_{y},\,{\vec{e}}_{z})$ stands for the unit vector. Substituting $\vec{A}$ into the Hamiltonian and assuming the environment (electromagnetic background) is very weak, we may expend the Hamiltonian up to the first order in $\vec{A}$,

2 $$\begin{array}{rcl}H & = & \vec{d}(\vec{k}-\frac{e}{\hslash }\vec{A})\cdot \vec{\sigma }+\sum _{n}{\omega }_{n}{b}_{n}^{\dagger }{b}_{n}\\  & \simeq  & \vec{d}(\vec{k})\cdot \vec{\sigma }-\frac{e}{\hslash }\sum _{j=x,y,z}(\nabla {d}_{j}\cdot \vec{A}){\sigma }_{j}+\sum _{n}{\omega }_{n}{b}_{n}^{\dagger }{b}_{n}\mathrm{.}\end{array}$$

Eq. (2) is the model Hamiltonian in this study. Straightforward calculations show that,

3 $$\begin{array}{l}H=\vec{d}(\vec{k})\cdot \vec{\sigma }+\sum _{n}{\omega }_{n}{b}_{n}^{\dagger }{b}_{n}+\sum _{n,j}{g}_{n}({b}_{n}+{b}_{n}^{\dagger })(\frac{\partial {d}_{j}}{\partial {k}_{x}}+\frac{\partial {d}_{j}}{\partial {k}_{y}}){\sigma }_{j},\end{array}$$

where ${b}_{n}({b}_{n}^{\dagger })$ is the annihilation (creation) operator of the boson mode with frequency *ω*_*n*_ and *g*_*n*_ is the coupling between the system and environmental mode *n*. The total Hamiltonian *H* contains the counter-rotating terms, and is not exactly solvable even for the simple cases of single mode or single excitation.

The eigenstates of *H*_0_ take

4 $$\begin{array}{ccc}|{\varepsilon }_{+}\rangle  & = & \cos \,\frac{\theta }{2}{e}^{-i{\phi }}|\uparrow \rangle +\,\sin \,\frac{\theta }{2}|\downarrow \rangle ,\\ |{\varepsilon }_{-}\rangle  & = & -\sin \,\frac{\theta }{2}{e}^{-i{\phi }}|\uparrow \rangle +\cos \,\frac{\theta }{2}|\downarrow \rangle \mathrm{.}\end{array}$$

*H* can be diagonalized by a unitary matrix *U*: $H^{\prime} ={U}^{\dagger }HU$,

5 $$\begin{array}{l}H{^{\prime} }\simeq |\vec{d}|{\tau }_{z}+\sum _{n}{\omega }_{n}{b}_{n}^{\dagger }{b}_{n}+\sum _{n}{g}_{n}({b}_{n}+{b}_{n}^{\dagger }){D}_{+}{\tau }_{+}+\sum _{n}{g}_{n}({b}_{n}+{b}_{n}^{\dagger }){D}_{-}{\tau }_{-},\end{array}$$

where we have omitted the term ${g}_{n}({b}_{n}+{b}_{n}^{\dagger }){\tau }_{z}$ because this term represents a energy shift to the system much smaller than the energy difference in *H*_0_^26^. Here, ${\tau }_{+}\equiv |{\varepsilon }_{+}\rangle \langle {\varepsilon }_{-}|$, ${\tau }_{-}\equiv |{\varepsilon }_{-}\rangle \langle {\varepsilon }_{+}|$ and ${\tau }_{z}\equiv |{\varepsilon }_{+}\rangle \langle {\varepsilon }_{+}|-|{\varepsilon }_{-}\rangle \langle {\varepsilon }_{-}|$, with $|\vec{d}|=\sqrt{{d}_{x}^{2}+{d}_{y}^{2}+{d}_{z}^{2}}$, $\cos \,\theta =\frac{{d}_{z}}{|\overrightarrow{d}|}$, $\tan \,{\phi }=\frac{{d}_{y}}{{d}_{x}}$, and

$$\begin{array}{ll}{D}_{+}= & (\frac{\partial {d}_{x}}{\partial {k}_{x}}+\frac{\partial {d}_{x}}{\partial {k}_{y}})(\cos \,{\phi }\,\cos \,\theta +i\,\sin \,{\phi })\\  & +(\frac{\partial {d}_{y}}{\partial {k}_{x}}+\frac{\partial {d}_{y}}{\partial {k}_{y}})(\sin \,{\phi }\,\cos \,\theta -i\,\cos \,{\phi })\\  & -(\frac{\partial {d}_{z}}{\partial {k}_{x}}+\frac{\partial {d}_{z}}{\partial {k}_{y}})\sin \,\theta ,\\ {D}_{-}\equiv  & {D}_{+}^{\ast }\mathrm{.}\end{array}$$

In the following, we will use the generalized version of the Fröhlich-Nakajima transformation^37–40^ to eliminate the high-frequency terms in the effective Hamiltonian: ${H}_{eff}={e}^{-S}H^{\prime} {e}^{S}$, with the operator *S*

6 $$\begin{array}{l}S=\sum _{n}{\lambda }_{n}{b}_{n}^{\dagger }{\tau }_{+}-\sum _{n}{\lambda }_{n}^{\ast }{b}_{n}{\tau }_{-},\end{array}$$

where *λ*_*n*_ will be determined later. The transformation can be done order by order, and the terms of order ${g}_{n}^{3}$ and higher will be ignored^38^. Up to the second order in the system-environment couplings, the effective Hamiltonian is given by,

7 $$\begin{array}{rcl}{H}_{eff} & = & {e}^{-S}H^{\prime} {e}^{S}\\  & \approx  & {H^{\prime} }_{0}+{H^{\prime} }_{I}+[{H^{\prime} }_{0},S]+[{H^{\prime} }_{I},S]+\frac{1}{2}[[{H^{\prime} }_{0},S],S],\end{array}$$

where ${H^{\prime} }_{0}$ and ${H^{\prime} }_{I}$ respectively take

8 $$\begin{array}{rcl}{H^{\prime} }_{0} & = & |\vec{d}|{\tau }_{z}+\sum _{n}{\omega }_{n}{b}_{n}^{\dagger }{b}_{n},\\ {H^{\prime} }_{I} & = & \sum _{n}{g}_{n}({b}_{n}+{b}_{n}^{\dagger }){D}_{+}{\tau }_{+}+\sum _{n}{g}_{n}({b}_{n}+{b}_{n}^{\dagger }){D}_{-}{\tau }_{-}\mathrm{.}\end{array}$$

By choosing the following form for *λ*_*n*_,

9 $$\begin{array}{l}{\lambda }_{n}=\frac{-{g}_{n}{D}_{+}}{\mathrm{2|}\vec{d}|+{\omega }_{n}},\end{array}$$

terms with ${b}_{n}^{\dagger }{\tau }_{+}$ and ${b}_{n}{\tau }_{-}$ can be eliminated from ${H^{\prime} }_{I}$. Noticing that ${H^{\prime} }_{I}$ contains only the rotating wave term, because the unitary transformation eliminate the counter-rotating terms, we write down the effective Hamiltonian *H*_*eff*_ as,

10 $$\begin{array}{l}{H}_{eff}=(|\overrightarrow{d}|+V){\tau }_{z}+\sum _{n}{\omega }_{n}{b}_{n}^{\dagger }{b}_{n}+\sum _{n}{g}_{n}{D}_{+}{b}_{n}{\tau }_{+}+\sum _{n}{g}_{n}{D}_{-}{b}_{n}^{\dagger }{\tau }_{-}+V,\end{array}$$

with $V=\frac{1}{2}{\sum }_{n}\frac{{g}_{n}^{2}{D}_{+}{D}_{-}}{\mathrm{2|}\overrightarrow{d}|+{\omega }_{n}}$. Here we have omitted the multiboson nondiagonal transition like ${b}_{n}^{\dagger }{b}_{n^{\prime} }^{\dagger }$ and ${b}_{n}{b}_{n^{\prime} }$, because the contributions of these nondiagonal terms to the physical quantities are $O({g}_{n}^{4})$ ^37,38^.

### The ground state of the whole system

Obviously, the effective Hamiltonian can be solved exactly, and the ground state is

11 $$\begin{array}{l}|g\rangle =|{\varepsilon }_{-}\rangle \otimes |\{{0}_{n}\}\rangle \mathrm{.}\end{array}$$

The ground state of the original Hamiltonian $|G\rangle$ is then given by $|g\rangle ={e}^{-S}|G\rangle$, leading to

12 $$\begin{array}{rcl}|G\rangle  & = & {e}^{S}|g\rangle \\  & = & \cos \,\lambda |{\varepsilon }_{-}\rangle \otimes |\{{0}_{n}\}\rangle +\sum _{n}{c}_{n}|{\varepsilon }_{+}\rangle \otimes |{1}_{n}\rangle ,\end{array}$$

where

$${c}_{n}=\,\sin \,\lambda \frac{{\lambda }_{n}}{\lambda },\,{\lambda }^{2}\equiv {\sum }_{n}{|{\lambda }_{n}|}^{2}={\sum }_{n}\frac{{g}_{n}^{2}{D}_{+}{D}_{-}}{{\mathrm{(2}|\vec{d}|+{\omega }_{n})}^{2}}.$$

In the continuum limit, the sum can be replaced by an integral. In the following we would consider the spectral density of the environment $J(\omega )={\sum }_{n}\,{g}_{n}^{2}\delta (\omega -{\omega }_{n})$ and a particular form

13 $$J(\omega )=\alpha \omega {(\omega /{\omega }_{c})}^{s-1}{e}^{-\omega /{\omega }_{c}}\mathrm{.}$$

Here *α* is the dimensionless coupling strength, the index *s* accounts for various physical situations. For example, the spectrum is sub-Ohmic when $s < 1$, and its is Ohmic when $s=1$ or super-Ohmic when $s > 1$. With these knowledge, we have

14 $$\begin{array}{l}{\lambda }^{2}=\int \alpha \omega {(\frac{\omega }{{\omega }_{c}})}^{s-1}{e}^{-\omega /{\omega }_{c}}\frac{{D}_{+}{D}_{-}}{{(\mathrm{2|}\vec{d}|+\omega )}^{2}}d\omega \mathrm{.}\end{array}$$

So far, we have presented a derivation for the ground state of a two-band model coupled to a multi-mode quantized field(or an environment) beyond the RWA.

## Results

### Hall conductance

In order to derive the Hall conductance as a response to a constant electric field $\vec{E}$, we consider the case that the electric field can be represented by a time-dependent vector potential, i.e., $\vec{k}(t)=\vec{k}-e\vec{E}t/\hslash$, and we take the field to be along the *x*-direction. This gives rise to a Hall current proportional and perpendicular to the electric field. The Hall conductivity, defined as the radio of the current density and the electric field, is therefore given by^41^

15 $$\begin{array}{rcl}\sigma  & = & \frac{{e}^{2}}{h}\int \frac{id{k}_{x}d{k}_{y}}{2\pi }[\langle \frac{\partial {\varepsilon }_{\nu }}{\partial {k}_{x}}|\frac{\partial {\varepsilon }_{\nu }}{\partial {k}_{y}}\rangle -\langle \frac{\partial {\varepsilon }_{\nu }}{\partial {k}_{y}}|\frac{\partial {\varepsilon }_{\nu }}{\partial {k}_{x}}\rangle ]\\  & \equiv  & C\frac{{e}^{2}}{h},\end{array}$$

where we use $|{\varepsilon }_{\nu }(\vec{k})\rangle$ to denote an eigenstate of the Hamiltonian with energy ${\varepsilon }_{\nu }(\vec{k})$ in the *v*-th magnetic Bloch band. The general relationship between the momentum-space topology and the quantization of physical responses has been discussed extensively by Volovik^42^. The Hall conductance is related to the Chern number *C*, and the Chern number *C* is a topological invariant, which is robust against a local deformation of the Hamiltonian and can only take an integer value. The Chern number could describe the topological property of the ground-state wave function, and then it is a measurable physical quantity^43,44^.

Substituting the ground state $|G\rangle$ into Eq. (15), we obtain

16 $$\begin{array}{ll}{\sigma }_{H}\,= & {\sigma }_{H}^{\mathrm{(0)}}+{\sigma }_{H}^{\mathrm{(1)}},\end{array}$$

where the zeroth- and first-order of ${\sigma }_{H}$ respectively take

17 $$\begin{array}{ll}{\sigma }_{H}^{\mathrm{(0)}}\,= & \frac{{e}^{2}}{h}\int \frac{id{k}_{x}d{k}_{y}}{2\pi }{{\rm{\Omega }}}_{-}(\vec{k}),\\ {\sigma }_{H}^{\mathrm{(1)}}\,= & \frac{{e}^{2}}{h}\int \frac{id{k}_{x}d{k}_{y}}{2\pi }\{{\sin }^{2}\lambda ({{\rm{\Omega }}}_{+}(\vec{k})-{{\rm{\Omega }}}_{-}(\vec{k}))\\  & -i{\sin }^{2}\frac{\theta }{2}(\frac{\partial {\cos }^{2}\lambda }{\partial {k}_{x}}\frac{\partial {\phi }}{\partial {k}_{y}}-\frac{\partial {\cos }^{2}\lambda }{\partial {k}_{y}}\frac{\partial {\phi }}{\partial {k}_{x}})\\  & -i{\cos }^{2}\frac{\theta }{2}(\frac{\partial {\sin }^{2}\lambda }{\partial {k}_{x}}\frac{\partial {\phi }}{\partial {k}_{y}}-\frac{\partial {\sin }^{2}\lambda }{\partial {k}_{y}}\frac{\partial {\phi }}{\partial {k}_{x}})\\  & +\sum _{n}(\frac{\partial {c}_{n}^{\ast }}{\partial {k}_{x}}\frac{\partial {c}_{n}}{\partial {k}_{y}}-\frac{\partial {c}_{n}^{\ast }}{\partial {k}_{y}}\frac{\partial {c}_{n}}{\partial {k}_{x}})\},\end{array}$$

with

$$\begin{array}{ll}{{\rm{\Omega }}}_{+}(\vec{k}) & =\,[\langle \frac{\partial {\varepsilon }_{+}}{\partial {k}_{x}}|\frac{\partial {\varepsilon }_{+}}{\partial {k}_{y}}\rangle -\langle \frac{\partial {\varepsilon }_{+}}{\partial {k}_{y}}|\frac{\partial {\varepsilon }_{+}}{\partial {k}_{x}}\rangle ],\\ {{\rm{\Omega }}}_{-}(\vec{k}) & =\,[\langle \frac{\partial {\varepsilon }_{-}}{\partial {k}_{x}}|\frac{\partial {\varepsilon }_{-}}{\partial {k}_{y}}\rangle -\langle \frac{\partial {\varepsilon }_{-}}{\partial {k}_{y}}|\frac{\partial {\varepsilon }_{-}}{\partial {k}_{x}}\rangle ]\mathrm{.}\end{array}$$

Here, ${{\rm{\Omega }}}_{-}(\vec{k})$ defines the Berry curvature of the lower bare band $|{\varepsilon }_{-}\rangle$. Hall conductance ${\sigma }_{H}$ in Eq. (16) for the two-band model subject to the environment has two different terms. The first term represents the linear response which returns to Eq. (15) when the system is closed, while the second one stands for a correction of the environment to the Hall conductance, and ${\sin }^{2}\lambda$ depends on *k*_*x*_, *k*_*y*_, the components of the Bloch vector.

Noticing that ${\sigma }_{H}$ in Eq. (16) can be considered as the Hall conductance under the influence of environment, which is derived through solving the ground state of the whole system including the effect of the counter-rotating terms on it. As ${\sigma }_{H}$ quantifies the response of the two-band model to a driving electric field, and the response is calculated by treating the system and environment on equal footing, we can claim that we have developed the other approach to study the response of open systems. This is different from the earlier studies base on the master equation, see for example^14^.

In the next subsection, we will present two examples that together exemplify the response of this quantum open system to the classical electric field.

### Examples

Consider a tight-binding model describing a magnetic semiconductor with Rashba-type spin-orbit coupling, spin-dependent effective mass, and a uniform magnetization on *z* direction^35^. This model can be described by Hamiltonian Eq. (1) with

18 $$\begin{array}{rcl}{d}_{x} & = & \sin \,{k}_{y},\\ {d}_{y} & = & \sin \,{k}_{x},\\ {d}_{z} & = & 2-\chi -\,\cos \,{k}_{x}-\,\cos \,{k}_{y}\mathrm{.}\end{array}$$

In a 2D band structure, the integral over the Brillouin zone (BZ) of the Berry curvature of the lower bare band is a topological invariant that is the well-known Chern number^5,43^. For the tight-binding model, when $0 < \chi  < 2$, the Chern number is 1, while for $2 < \chi  < 4$, the Chern number is −1, and for $\chi  < 0$ and $\chi  > 4$, the Chern number is 0.

**Figure 1:**
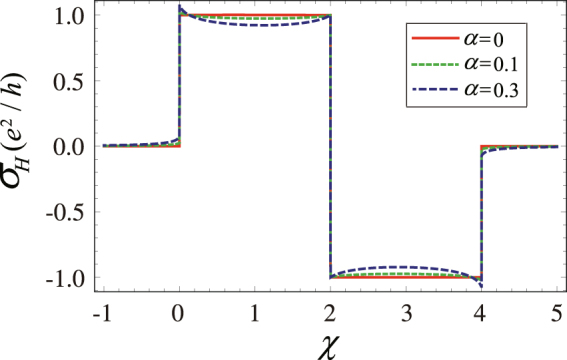
*σ*_*H*_ (in units of ${e}^{2}/h$) as a function of *χ* in the tight-binding model with ${d}_{x}=\,\sin \,{k}_{y},\,{d}_{y}=-\sin \,{k}_{x},$ ${d}_{z}=2-\chi -\,\cos \,{k}_{x}-\,\cos \,{k}_{y}$. For comparison, the conventional Hall conductance (the red solid line) is also shown, corresponding to the coupling strength $\alpha =0$. The other parameters chosen are $s=1$ and ${\omega }_{c}=1$.

The Hall conductance ${\sigma }_{H}$ (in units of ${e}^{2}/h$) defined in Eq. (16) as a function of *χ* is shown in Fig. 1. The features observed from Fig. 1 demonstrate that the phase transition points, i.e., *χ* = 0, 2, 4, remain unchanged. The Hall conductance may not be an integer due to the presence of environment, the topological phase transition, however, survives in the environment. In contrast with the well-known Hall conductance shown in Fig. 1 (red sold line), ${\sigma }_{H}$ is not a constant in regions, $0 < \chi  < 2$, $2 < \chi  < 4$, $\chi  < 0$ and $\chi  > 4$, which results from the correction ${\sigma }_{H}^{\mathrm{(1)}}$ in Eq. (16). Physically, the correction comes from the counter-rotating terms, which would mix the two bands and then leads to a different response to the field. We also find that at the critical point *χ* = 0, the Hall conductance ${\sigma }_{H}$ increases compared to the traditional Hall conductance ${\sigma }_{H}^{\mathrm{(0)}}$, while it decreases at *χ* = 4. Except at these critical points, the Hall conductance almost always decreases due to the environment.

**Figure 2:**
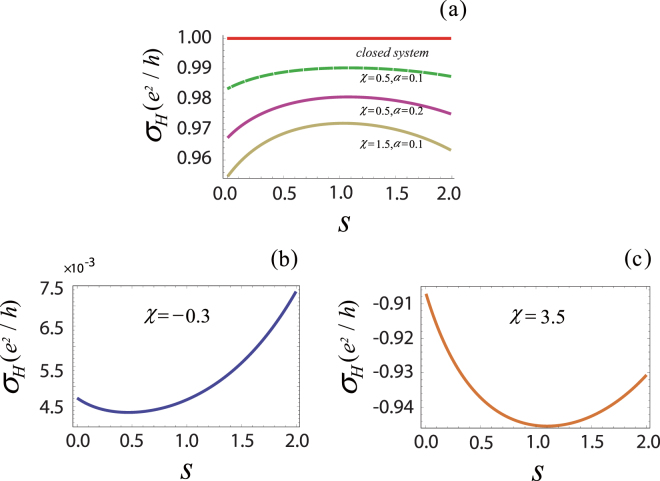
${\sigma }_{H}$ (in units of ${e}^{2}/h$) for various index *s* of the spectral density is plotted as a function of *s*. In this plot, ${d}_{x}=\,\sin \,{k}_{y},\,{d}_{y}=\,\sin \,{k}_{x},\,{d}_{z}=2-\chi -\,\cos \,{k}_{x}-\,\cos \,{k}_{y}\mathrm{.}$ (**a**) When we choose $\chi =0.5$ and $\chi =1.5$ respectively, the Hall conductance at different coupling strength $\alpha =0.1$ or $\alpha =0.2$ of the quantized field compares with the integer Hall conductance (red solid line). The other parameters chosen are $\alpha =0.2$ and ${\omega }_{c}=1$ for (**b**) $\chi =-0.3$ and (**c**) $\chi =3.5$.

In Fig. 2, we numerically show the Hall conductance against *s* for various spectral density characterized by the parameter *χ*, and the environment can change the topology of the system. The features found from Fig. 2 also support the conclusion that the topological phase transition survives for the two-mode system in environments.

The two-band model used in this paper describes a TR symmetry-breaking system^35^. The Hall conduction of the system is quantized and it is determined by the first Chern number of the Berry phase gauge field in the BZ^5,12^. Theories of topological materials are often formulated using tight-binding models as we used in this paper, which can be simulated by ultra-cold atomic gases^45^ in optical lattices. Besides, scanning tunnelling microscopy (STM) can also be used to fabricate and characterize lattice structures with atomic precision in the solid state. Very recently, it has been shown that the tight-binding model can be realized in a vacancy lattice on the ${\rm{Cl}}/\mathrm{Cu}(\mathrm{100})$ substrate surface^46^.

**Figure 3:**
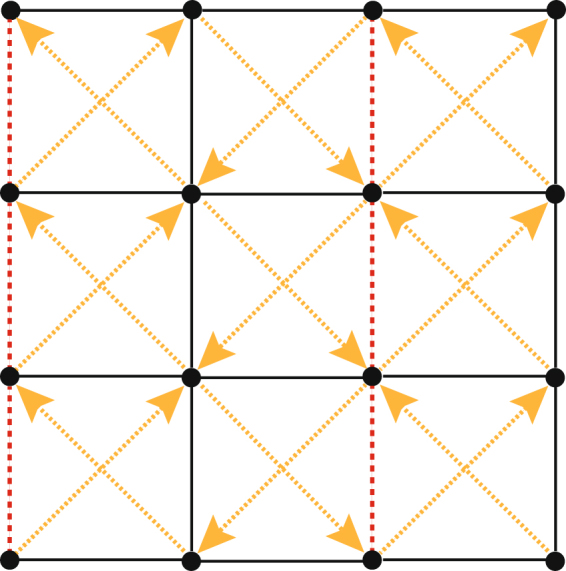
The hopping amplitudes of the Hamiltonian: (1) black solid links *t*_1_, (2) red dashed links −*t*_1_, (3) diagonal links *it*_2_ in the direction of the arrow.

In the second example, we consider a two-dimensional model with a tight-binding Hamiltonian for electron gas on a square lattice^47–49^,

19 $$\hat{H}=\sum _{lj}{t}_{lj}{e}^{i{{\varphi }}_{lj}}{c}_{l}^{\dagger }{c}_{j}+\sum _{l}{\varepsilon }_{l}{c}_{l}^{\dagger }{c}_{l},$$

where ${c}_{l}$ and ${c}_{l}^{\dagger }$ are respectively the fermion annihilation and creation operators at the lattice site ${\overrightarrow{r}}_{l}$, respectively. ${\varepsilon }_{l}$ is an on-site chemical potential. ${t}_{lj}$ describes the hopping amplitude of electron from site *l* to its nearest neighbor site *j*. ${t}_{lj}={t}_{1}$, ${{\varphi }}_{l,l+\hat{x}}=0$ and ${{\varphi }}_{l,l+\hat{y}}=\pi$. When the model is for the case where the sites *l* and *j* are next-nearest neighbors, ${t}_{lj}={t}_{2}$ and ${{\varphi }}_{l,l+\hat{x}+\hat{y}}={{\varphi }}_{l+\hat{x},l+\hat{y}}=\pi$, see Fig. 3.

Here, we will choose a two-site unit cell $(l,\,l+\hat{x})$ and rewrite the Hamiltonian as $\hat{H}={\sum }_{\overrightarrow{k}}{\psi }_{\overrightarrow{k}}^{\dagger }(\varepsilon -2{t}_{1}\hat{H}(\vec{k})){\psi }_{\overrightarrow{k}}$, with ${\psi }_{\overrightarrow{k}}={({c}_{l},{c}_{l+\hat{x}})}^{{\rm{T}}}$. $\hat{H}(\vec{k})$ then takes the form of Eq. (1) with ${d}_{x}=-\cos \,{k}_{x}$, ${d}_{y}=m\,\sin \,{k}_{x}\,\sin \,{k}_{y}$, ${d}_{z}=\,\cos \,{k}_{y}$, and $m=2{t}_{2}/{t}_{1}$. Recall that the Chern number^5,43^ of this system is 1(−1) for $m > \mathrm{0(} < \mathrm{0)}$ ^50^.

**Figure 4:**
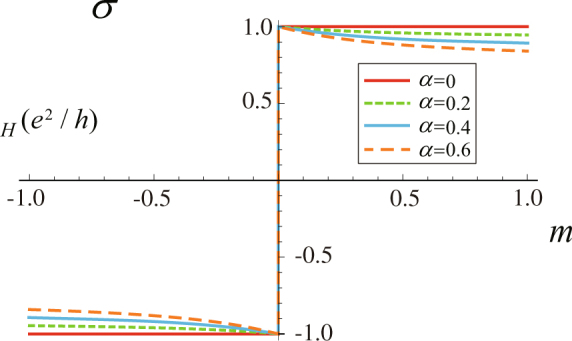
${\sigma }_{H}$ (in units of ${e}^{2}/h$) as a function of *m* at various coupling strength *α* in Eq. (13). In this model, ${d}_{x}=-\cos \,{k}_{x}$, ${d}_{y}=m\,\sin \,{k}_{x}\,\sin \,{k}_{y}$, ${d}_{z}=\,\cos \,{k}_{y}$. Here, the range of the parameter *m* is in $[-\mathrm{1,}\,\mathrm{1]}$. The other parameters, $s=1$ and ${\omega }_{c}=1$ have been used in this plot.

In Fig. 4, we plot ${\sigma }_{H}$ as a function of *m* for the different values of the coupling strength *α*. Note that both the Hall conductance *σ* in the closed system and ${\sigma }_{H}$ in the open system change the sign when *m* crosses zero, see Fig. 4, which shows explicitly that the phase transition point for the closed and open system is at $m=0$. This suggests that the topological phase transition survives in the open system considered here. We find that the environment suppresses the Hall conductance ${\sigma }_{H}$, but it does not change the phase transition points, see Fig. 4. With the increasing of *α*, the effect of environment on the Hall conductance becomes evident.

The topological invariant was introduced to explain phase transitions that are beyond the conventional framework of symmetry breaking. Clearly, taking the environment into account, the Hall conductance (equivalently the topological invariant–Chern number) may not be an integer. This will not be an obstacle to use the Hall conductance to detect topological properties of insulator states for quantum open system, since the phase transition points are still there. Vatsal Dwivedi and Victor Chua constructed a generalized transfer matrix corresponding to noninteracting tight-binding lattice models, which can subsequently be used to compute the bulk bands as well as the edge states^51^. The Hall conductance as a function of the gap parameter in the post-quench Hamiltonian displays a universal nonanalytic behavior in a generic two-band Chern insulator^13^, such as the Dirac model, the Haldane model, or the Kitaev honeycomb model in the fermionic basis^44^. Though it is difficult to observe quantum transport of the surface states, which are usually covered by bulk carriers caused by material defects. Recently, several explicit experimental observations of QHE based on surface states have been obtained in topological materials^52–55^. These experimental schemes may provide us with a platform for realizing the prediction in this paper.

## Discussion

We have studied the Hall conductance of two-band system in the presence of multi-mode quantized field. We can recover the traditional Hall conductance ${\sigma }_{H}^{\mathrm{(0)}}$ in Eq. (16) for the two-band model in closed system, whereas it can not return to the usual Hall conductance when the environment is taken into account. We treat the first-order of Hall conductance ${\sigma }_{H}$ as the correction. This correction comes from the counter-rotating terms, which would mix the two bands and then leads to a different response to the external field. The Hall conductance for open system can not be written as a multiple of a Chern number and a constant, or as a weighted sum of Chern numbers, in this paper, there is no topological invariant for open systems.

It is well known that the change of the topology in the ground state of a system is accompanied by a topological phase transition. In the celebrated paper by Thouless^5^, the Hall conductance (equivalently the Chern number) can be probed, and the Chern number usually keeps invariant as the Hamiltonian changes. The Chern number then has a jump, which indicates a topological phase transition in the ground state^43^.

In summary, we find that the jump remains unchanged, but the Hall conductance is suppressed, when a two-band system is in contact with an environment modeled by a multi-mode quantized field. The calculation is based on the Kubo formula by treating the system and environment on equal footing. We do not use the rotating wave approximation in obtaining an effective Hamiltonian and the ground state of the whole system. This is different from the earlier study, where the starting point is the master equation for the open system within the RWA. This result suggests that the transition points of topological phase are robust against the environment. Although the analysis has been restricted to the two-band model, we believe that the observation makes the topological materials immune to the influence of the quantum field, and then supports its application into quantum optics and condensed matter physics.
